# Estimating Lower Extremity Running Gait Kinematics with a Single Accelerometer: A Deep Learning Approach

**DOI:** 10.3390/s20102939

**Published:** 2020-05-22

**Authors:** Mohsen Gholami, Christopher Napier, Carlo Menon

**Affiliations:** 1 Menrva Research Group, Schools of Mechatronic Systems Engineering & Engineering Science, Simon Fraser University, Metro Vancouver, BC V5A 1S6, Canada; mohsen_gholami@sfu.ca (M.G.); cnapier@sfu.ca (C.N.); 2 Department of Physical Therapy, University of British Columbia, Vancouver, BC V6T 1Z4, Canada

**Keywords:** accelerometer, inertial sensors, wearable sensors, gait monitoring, kinematic, running, convolutional neural networks

## Abstract

Abnormal running kinematics are associated with an increased incidence of lower extremity injuries among runners. Accurate and unobtrusive running kinematic measurement plays an important role in the detection of gait abnormalities and the prevention of injuries among runners. Inertial-based methods have been proposed to address this need. However, previous methods require cumbersome sensor setup or participant-specific calibration. This study aims to validate a shoe-mounted accelerometer for sagittal plane lower extremity angle measurement during running based on a deep learning approach. A convolutional neural network (CNN) architecture was selected as the regression model to generalize in inter-participant scenarios and to minimize poorly estimated joints. Motion and accelerometer data were recorded from ten participants while running on a treadmill at five different speeds. The reference joint angles were measured by an optical motion capture system. The CNN model predictions deviated from the reference angles with a root mean squared error (RMSE) of less than 3.5° and 6.5° in intra- and inter-participant scenarios, respectively. Moreover, we provide an estimation of six important gait events with a mean absolute error of less than 2.5° and 6.5° in intra- and inter-participants scenarios, respectively. This study highlights an appealing minimal sensor setup approach for gait analysis purposes.

## 1. Introduction

The kinematics of the lower extremity are an important area of research in human running gait analysis. Abnormal running kinematics are associated with an increased incidence of lower extremity injuries among runners [1,2]. While injuries may occur due to deviations in any plane, two of the most common kinematic patterns associated with injured runners occur in the sagittal plane (greater knee extension and ankle dorsiflexion at initial contact [2]). Correcting abnormal kinematics has been suggested to reduce the risk of injury [3,4]. Runners’ gait parameters can also change during a prolonged run due to fatigue [5,6,7], which may increase injury risk as the runner deviates from their normal gait pattern [8,9]. Reducing injury risk factors via altering running biomechanics has been demonstrated to be feasible in a lab environment [4,10], but this requires sophisticated equipment and software. Providing this feedback in an ecologically valid (in-field) context requires the ability to both measure and give feedback using wearable devices. This can be done currently only by using simple metrics such as cadence [11] or peak tibial acceleration [12], but the measurement of joint kinematics is much more complex. Developing an accurate, unobtrusive gait monitoring wearable system to measure running kinematics is still subject to research.

In contrast to optical motion-capture-based gait analysis, wearable sensors enable continuous and unobtrusive gait monitoring during in-field activity. Inertial measurement units (IMUs) have been widely employed as a portable system for the estimation of human gait kinematics and kinetics [13,14]. Consisting of an accelerometer, gyroscope, and magnetometer, IMUs measure the orientation of body segments by sensor data fusion.

### 1.1. IMU-Based Gait Kinematic Estimation

Inertial-based sensors have been employed to measure human kinematics based on three main approaches: (1) orientation-based methods that entail calculating the relative orientation of two adjacent segments based on the orientation of IMUs mounted on the distal and proximal segments; (2) model-based methods that take advantage of kinematic constraints; and (3) data-driven methods that employ supervised learning models to estimate reference joint angles.

The orientation-based method has traditionally been used to compute human kinematics [13,15] but has some limitations, including errors in sensor-to-segment alignment. The anatomical orientation of the bone is different from the local reference frame of the IMUs, requiring a calibration step to calculate the relative orientation of the IMU and the anatomical reference frame. However, the precision of the calibration also relies on performing predefined movements correctly, which may not be reliable, especially in injured populations [15,16]. Previous studies have used motion capture or RGB cameras to calculate the relative orientation of IMUs and the anatomical position before testing [17,18,19,20]. Although this method obtains lower errors, it requires prior calibration with cameras for each new session or participant. Another limitation of the orientation-based method is the drift in sensor signal that is due to signal integration operation, which is usually an essential step. Sensor data fusion using the Kalman filter is the main approach that has been proposed to overcome the drift problem [21]. However, the signal drift in noisy environments with ferromagnetic disturbances and during prolonged data recording is still problematic [13].

Kinematic or musculoskeletal model-based approaches consider the kinematic constraints of the human body to overcome the limitations of sensors such as drift and sensor-to-segment alignment. Optimization is driven to reduce the error between the orientation of the IMUs and body segments by taking into account the anatomical constraints of the human body [22]. Dorschky et al. [23] proposed estimating lower extremity joint angles using raw accelerometer and gyroscope data by solving optimization problems based on the musculoskeletal dynamics model introduced by van den Bogert et al. [24]. Joint constraints have also been proposed to overcome sensor-to-segment calibration [16].

The third method for lower extremity joint angle monitoring is a data-driven approach that relies on machine learning algorithms [25,26,27]. This approach feeds the raw signal [26] or orientation calculated by filtering methods into a machine learning model [27]. The machine learning model takes care of the sensor-to-segment calibration and calculates the joint angles. The main limitation of the data-driven method is the dependency on the supplied dataset and generalizability to other individuals or populations. Wouda et al. [27] used three IMUs to measure ground reaction forces and knee joint angles in running based on a neural network model. They demonstrated the potential of data-driven methods to estimate both kinematic and kinetic parameters during highly dynamic movement. Previous studies have reported root mean squared error (RMSE) values for sagittal plane lower extremity joint angles in walking ranging from 1.5° to 11° [15,28,29], while in running a range of 3.4°–13° for hip, knee, and ankle angle estimation has been reported [17,27]. When comparing the findings of previous studies, it is important to mention that many studies have included prior information for the model, such as a participant-specific optimized model [23], sensor-to-segment calibration [17,19,20,30], or sensor initial orientation on the body segment [20,31].

### 1.2. Reducing Number and Degrees of Freedom of IMUs:

Mounting an IMU on each segment for lower extremity kinematic estimation requires seven IMUs to provide full lower extremity joint angle measurement. This approach is cumbersome and obtrusive. Furthermore, it is unrealistic to expect a user at the consumer level to attach multiple IMUs due to the increased cost, reduced practicality, and probability of fixation error. Hence, there is a trend to reduce the number of required sensors for joint angle estimation. There are two approaches proposed so far to reduce the number of sensors: (1) model-based and (2) data-driven.

A model-based approach was proposed by Hu et al. [32] using four IMUs only to estimate the hip, knee, and ankle angles based on a serial chain model and solving the inverse kinematics. Bonnet et al. [31] used a single IMU on the shank to measure the hip and knee angles during several rehabilitation exercises by considering the mechanical constraints between proximal and distal segments. These methods demonstrated a greater error compared to a comprehensive sensor setup or were introduced for specific tasks. Data-driven methods have also been proposed for human full-body motion monitoring [33,34] and lower extremity walking analysis [35] with a reduced number of sensors. Recently, Lim et al. [35] proposed using a single IMU on the pelvis close to the center of mass to measure the lower limb kinematics and kinetics in walking. However, the application of a reduced number of inertial sensors to measure gait kinematics during a highly dynamic motion such as running has not yet been investigated.

### 1.3. Other Considerations

One issue with the use of IMUs for joint orientation measurement is the sensitivity of the magnetometer to ferromagnetic disturbances. For this reason, there is a trend towards magnetometer-free inertial-based systems for joint angle monitoring. Power consumption is also a significant concern during activities that occur over a prolonged period. Long distance running can have a duration of up to two hours or more. When comparing the power consumption of accelerometers and gyroscopes, the former has the advantage of a lower power consumption [36]. Therefore, the use of an IMU consisting of an accelerometer alone has the advantage of avoiding ferromagnetic disturbances and lasting throughout longer-duration activities.

The anatomical location of the IMU impacts the practicality of its use as well as the quality of the data. Rigid fixation of IMUs on the shank or thigh is less practical and more difficult than using a shoe-mounted system. The fixation that can be applied using a shoe- or lace-mounted IMU is likely to lead to more reliable data.

The ability to measure running kinematics outside of the lab and in real-world environments has the potential not only to allow researchers to study the impact of different terrain and fatigue states on running biomechanics, but also to prevent injuries when paired with real-time biofeedback [37]. In order to achieve this goal, accurate and unobtrusive monitoring of kinematics needs to be demonstrated in a controlled setting. In this study, we aimed to investigate the performance of a single shoe-mounted accelerometer to monitor lower extremity running kinematics in the sagittal plane. A secondary aim was to reduce the degrees of freedom of the IMU. A data-driven approach is presented based on convolutional neural networks. The performance of the method in inter- and intra-participant scenarios has been evaluated.

## 2. Materials and Methods

### 2.1. Experiment Setup

**Figure 1:**
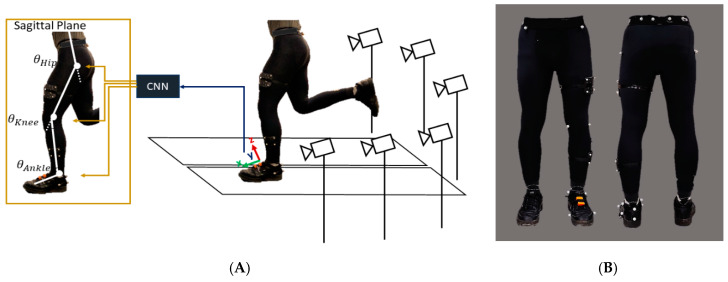
(**A**) Experimental setup including six motion capture cameras and a split-belt treadmill. The schematic of angles estimated using the raw signal of a foot-mounted accelerometer. (**B**) Reflective marker positions on the lower extremity.

Twenty-five reflective markers were affixed to each participant prior to testing, and a static calibration trial was initially collected to form a musculoskeletal model based on Napier et al. (Figure 1) [38] using a 6-camera motion analysis system (Vicon, Oxford, UK). Ten static/calibration markers (anterior superior iliac spines, greater trochanters, left medial/lateral femoral condyles, left medial/lateral malleoli, and left first and fifth metatarsal heads) were removed following the static trial, and the 15 remaining markers (posterior superior iliac spines, iliac crests, clusters of 4 on the thigh and shank, and a triad on the heel) were by definition tracking and calibration markers, as they were on for both static and dynamic trials. The ankle joint center was identified as the midpoint between the 2 ankle malleoli markers; the knee joint center was identified as the midpoint between the 2 femoral condyle markers, and the hip joint center was identified using the method of Bell et al. [39].

The acceleration of the foot was measured by an Xsens inertial measurement unit (MTw Awinda, Xsens, Enschede, The Netherlands) mounted on the shoe, as shown in Figure 1. The Xsens unit has an accelerometer, gyroscope, and magnetometer; however, in this study only the raw data of the accelerometer were used for data processing. Both the motion capture and accelerometer data were recorded at a 100 Hz sampling rate. The accelerometer and motion capture data were synchronized using an analog signal from each of the Vicon and Xsens units.

### 2.2. Data Collection

Ten healthy male Caucasian participants (age 27 ± 4 years, height 177 ± 7 cm, and weight 72 ± 7 kg) participated in this study. The number of participants (sample size) was conducted in G*Power 3.1.9.3 to detect a strong (R^2^ > 0.80) association between our method and the gold standard optical motion capture method for computing joint kinematics. To obtain 80% power to detect significant (*p* < 0.05) associations, we determined that 10 participants were required. Sex was not expected to influence our results [40]. The study protocol was approved by the Office of Research Ethics at Simon Fraser University, and all participants provided informed consent.

The data recording protocol consisted of 15 trials of running at five different speeds—8, 9, 10, 11, and 12 km/h—with three trials of 60 s at each speed. The participants were given time to warm up and familiarize themselves with the treadmill before data recording started from the slowest speed. The participants were given a short break after each trial.

### 2.3. Data Preprocessing

**Figure 2:**
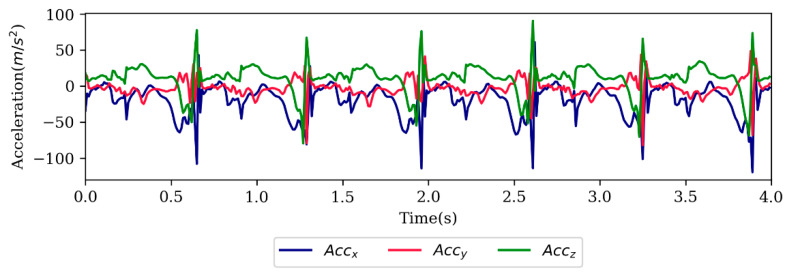
Sample of raw accelerometer signal.

The raw accelerometer data from all three axes were recorded by Xsens software and filtered using the SciPy Python library [41]. The accelerometer data were not normalized or standardized. A total of 6% of the dataset was excluded due to the asynchronized accelerometer and motion signals. The motion capture data were considered the gold standard reference for the kinematic data for this study. The marker trajectories were imported to Visual 3D software (C-Motion, Inc., Germantown, MD, USA) and the joint angles were computed and filtered with Visual 3D. The motion and accelerometer data were filtered by a fourth-order Butterworth low-pass filter with a cut-off frequency of 6 Hz following the recommendation of previous studies [42]. A sample of raw accelerometer data is shown in Figure 2.

### 2.4. Deep Learning Model

Convolutional neural networks (CNNs) have been recently applied to different signal processing problems and have also shown promising results in human motion estimation using wearable sensors [43]. In this study, a one-dimensional CNN(1D-CNN) was implemented in Keras blackened TensorFlow [44]. The input and output of the model were as follows:

(1) $$\left[\theta_{hip},\theta_{knee},\theta_{ankle}\right]=h\left(X\right)$$

where the CNN model is denoted with h and *X* was a matrix of the shoe-mounted accelerometer’s data with a shape of 60×4:

(2) $$X^{T}=[\begin{array}{cc} a_{1x}{}^{t-n+1} & a_{1x}{}^{t-n+1}\\ a_{1y}{}^{t-n+1} & a_{1y}{}^{t-n+1}\\ a_{1z}{}^{t-n+1} & a_{1z}{}^{t-n+1}\\ a_{1xyz}{}^{t-n+1} & a_{1xyz}{}^{t-n+1} \end{array}\cdots \begin{array}{c} a_{1x}{}^{t+n}\\ a_{1y}{}^{t+n}\\ a_{1z}{}^{t-n}\\ a_{1xyz}{}^{t-n} \end{array}]$$

A time window was moved over the signal with a length of 2*n* = 600 ms that covered equal samples of the past and the future time steps. $a_{x},\;a_{y},$ and $a_{z}$ were acceleration in three different axes. $a_{xyz}$ was the root sum squared of the acceleration with the following formula:

(3) $$a_{xyz}=\sqrt{a_{x}{}^{2}+a_{y}{}^{2}+a_{z}{}^{2}}.$$

The $a_{xyz}$ is axis free and is less prone to changes in the IMU’s orientation on body segments from person to person.

The 1D-CNN model had four convolutional (Conv) layers and a max-pooling layer. The number of features at the first and second two Conv layers was 50 and 100, respectively. The architecture and layer shapes are summarized in Table 1. All the trainable layers were initialized with a Xavier normal initializer. The Conv and fully connected layers were activated with a rectified linear unit, while the output layer was activated with a linear function. The kernel size and stride values were selected to be 3 and 1, respectively. The parameters were optimized by an Adam optimizer with a learning rate of 0.001. The batch size for training the model was selected to be 512 and the number of epochs was fixed to 50. A dropout layer was defined as the first layer for inter-participant scenarios to help the model generalize while testing on a new participant. The dropout layer was not defined for intra-participant models. Since the errors of hip and ankle angle estimation were greater than the knee, a customized loss function was defined to improve the ankle and hip estimated joint angles as follows:

(4) $$Loss=\frac{1}{3}\left(A\times RMSE\left(\theta_{Hip}\right)+B\times RMSE\left(\theta_{Knee}\right)+C\times RMSE\left(\theta_{Ankle}\right)\right),\;\;\;\;RMSE=\overset{n}{\underset{i=1}{\sum }}{\left(y-\hat{y}\right)}^{2}$$

where *A*, *B*, and *C* are weight parameters selected empirically to be 3, 1, and 3, respectively.

### 2.5. Evaluation Methods

**Figure 3:**
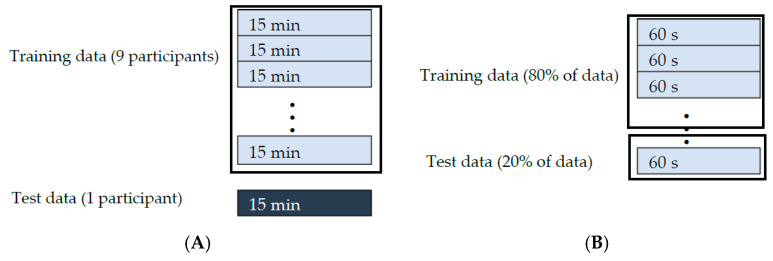
Splitting data for training and testing for (**A**) inter-participant method and (**B**) intra-participant method.

Two evaluation methods were considered: (1) intra-participant and (2) inter-participant. In the intra-participant method, CNN models were trained and tested using the running data of each participant. Running trials of each participant were randomly concatenated and then 80% of data was selected for training and the remaining 20% for the test. In the inter-participant method, the CNN models were trained on the data of nine participants and tested on the tenth participant, and this continued until all the participants were assigned to the test set. Figure 3 shows a schematic of splitting the data into training and test following a-leave-one-person-out cross-validation scheme for inter-participants In the intra-participants scenario (Figure 3), data were split into train and test regardless of the speeds and consequences of trials.

**Figure 4:**
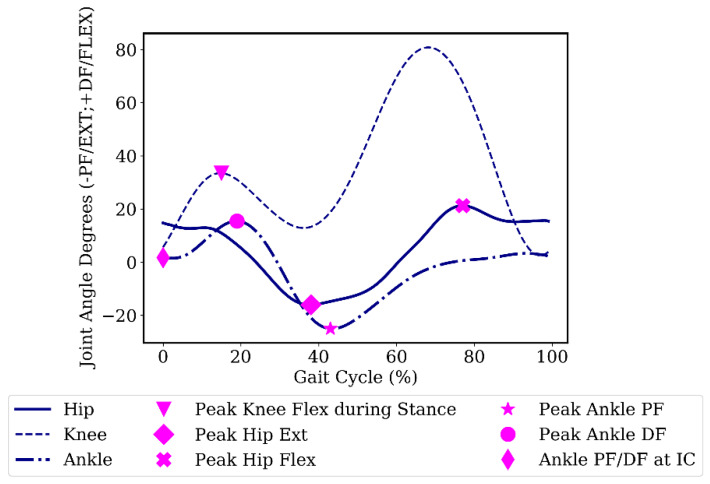
Gait events over a sample participant’s gait cycle. Flexion (Flex)/Dorsiflexion (DF) are positive; extension (Ext)/Plantarflexion (PF) are negative. IC, initial contact.

We also investigated the error of the estimated angles at six important gait events. The selected gait events were: peak knee flexion angle during stance phase, peak hip flexion/extension angle, peak ankle plantar/dorsiflexion angle, and ankle plantar/dorsiflexion angle at initial contact. Figure 4 shows the specified gait events in the gait cycle. Increasing the knee flexion angle during the stance phase has been suggested to attenuate shock [45]. The ankle angle at initial contact is also representative of the foot-strike pattern, which has relevance to running injuries. The initial contact during running was determined using the minimum vertical height of the heel markers for each stride [46]. The remaining gait events were detected using peak detection of the reference joint angles.

Three different evaluation metrics were considered in this study: (1) root mean squared error (RMSE), (2) normalized root mean squared error (NRMSE), and (3) root mean squared values (R^2^). The first two evaluation metrics reflect the measurement error, while the third evaluation metric reflects the goodness of predicted values. To calculate the NRMSE, the RMSE was divided by the range of angles in the test dataset. Equations of RMSE and NRMSE are as follows:

(5) $$RMSE=\overset{n}{\underset{i=1}{\sum }}{\left(y-\hat{y}\right)}^{2},\;\;\;\;\;NRMSE=\frac{RMSE}{y_{max}-y_{min}}$$

## 3. Results

### 3.1. Intra-Participant Models

The average root mean squared value (R^2^) for ten participants for hip, knee, and ankle angles was greater than 0.97, while the RMSE and NRMSE were lower than 3.4° and 4.6%, respectively. The average intra-participant estimations among the ten participants are shown in Table 2.

The estimated and reference discrete angles among the participants and the mean absolute error (MAE) of estimated angles are reported in Table 3. The mean difference between the estimated and reference angles for all joints was less than 1°. However, the mean absolute error of the estimated values was up to 2.4 degrees.

### 3.2. Inter-Participant Models

In this section, the CNN model was trained on 9 participants and then tested on the 10th participant. The error of the estimated angles in the inter-participant scenario was less than 6.5°, with average R^2^ values of 0.84, 0.93, and 0.73 for hip, knee, and ankle, respectively (Table 4). The estimated RMSE was normalized with a range of angles for each participant and the average NRMSE between the 10 participants was less than 11% for the hip, knee, and ankle.

The estimated and reference discrete values for the inter-participant method are shown in Table 5. The mean difference between the estimated and reference values was less than 4°, with peak knee flexion during stance and peak hip flexion displaying the greatest mean absolute error.

**Figure 5:**
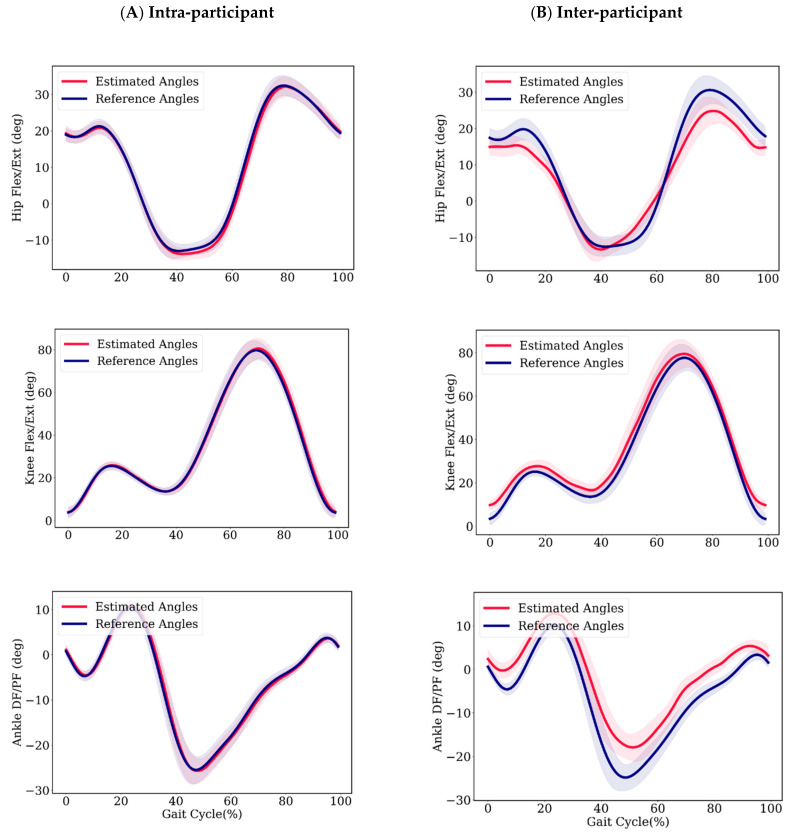
Average estimated and reference angles of participant 7 with standard deviations in shadow for (**A**) intra-participant model and (**B**) inter-participant model. Flexion (Flex)/Dorsiflexion (DF) are positive; extension (Ext)/Plantarflexion (PF) are negative.

Figure 5 shows the average estimated and reference joint angle trajectories for participant number 10, with the standard deviation (shaded region) of estimated and reference angles for all participants. The estimated angles follow a similar trajectory to the reference angles.

## 4. Discussion and Conclusions

In this study, we investigated the performance of a single shoe-mounted accelerometer for hip, knee, and ankle joint angle estimation. To the best of our knowledge, this is the first study that has used a single accelerometer for lower extremity joint angle estimation. A convolutional neural network was used to estimate joint angles in inter- and intra-participant scenarios. The accelerometer was placed on the shoe (lace-mount), which is a convenient position for runners and the most common site for consumer-grade IMUs in runners [47]. Mounting IMUs on the thigh and shank is less practical for runners, and IMUs mounted on the waist can be affected by the movements of both legs. Moreover, mounting accelerometers on the shank, thigh, or waist increases the likelihood of inflated values due to improper fixation or, in the case of the thigh, soft tissue artefact. The study in larger scope recommends the potential of deep learning-based approaches for inertial measurement unit data processing to tackle accurate human motion monitoring and reduce the number of sensors required.

In both inter- and intra-participant scenarios, the knee joint angle was better estimated than the hip and ankle joint angles. We reason that the lower error of the knee angle estimation was due to consistent knee angle trajectories among participants. Moreover, the degrees of freedom of the knee angle is lower compared to the ankle and hip joints. The ankle and hip have greater movements available to them in the frontal and transverse planes that may be obscured when we are only considering sagittal plane movements.

In the inter-participant scenario, the error of the ankle joint angle estimation was significant, with an NRMSE of 11% and an R^2^ of 0.78. We hypothesize that the greater variation in ankle joint angle throughout the gait cycle among participants was the reason for the significant ankle angle estimation error. The average standard deviations of joint angles through the gait cycle divided by the range of motion over ten participants were 0.09, 0.07, and 0.12 for the hip, knee, and ankle, respectively. Although there is an offset between the estimated ankle angle and the reference values in Figure 5, the estimated values are close to the reference value at initial contact (<1°). The ankle angle at initial contact is an important measure, since it can be used to determine the foot strike pattern of the individual [48]. This value was estimated with a mean absolute error of less than 3˚ in the inter-participant method.

In this study, the accuracy of this model at six key gait events was reported. All discrete values in the inter-participant evaluation were underestimated and the peak knee flexion estimation had the greatest difference from the reference value. For biomechanical analysis, the accuracy of the estimated angles at key gait events has importance in relation to injury risk and performance variables. Changes in these variables over time (throughout a prolonged run, for instance) may indicate the runner’s level of fatigue [5,6,7] and therefore the risk of developing injury [1,2,9]. A portable system that could accurately monitor the joint angles at key gait events over the course of a prolonged run, therefore, could be a valuable tool to prevent running-related overuse injuries.

The error of estimated angles in the intra-participant method was small, with an RMSE ranging between 1.8° and 3.4°. However, errors were greater for the inter-participant evaluation method (4.7°–6.5°). The reported error is in comparison to an optical motion capture system. While optimal motion capture is considered the gold standard measurement tool for gait analysis, these systems have been reported to have an error of 0.48°–7.36° in the sagittal plane hip, knee, and ankle measurement between two different sessions [49]. This error is mainly due to inaccurate marker placement and the marker-positioning error of the system during dynamic movement. The optical system’s error may propagate to the inter-participant estimated angles. In this study, one person placed markers on participants’ anatomical landmarks to minimize the inter-participant error of reference joint angles.

In comparison with previous studies, Wouda et al. [27] used three IMUs to estimate the knee joint angle and obtained an average error of ~4° and <13° in intra- and inter-participant evaluation methods, respectively. Previous studies have reported a smaller error in static movements compared with dynamic movements [15], since the range of motion, noise, and sensor movement are greater during activity. Table 6 compares previous studies that have reported inertial-based methods for joint angle measurement during running. The number of sensors, whether the method is intra/inter-participant, the computation methods, the number of joint angles that were measured, and the error of measurement are compared. Intra-participant methods require some input from the participant, such as initial sensor orientation on the segment [20] or a sample of reference gait kinematics during running [23,27] that requires motion capture data collection, which is not available to most runners, coaches, or clinicians. Our study used only a single inertial sensor with only accelerometer data. We also had fewer sensor degrees of freedom and a lower error in the inter-participant scenario thanks to the deep convolutional neural networks.

## 5. Limitations and Future Work

The generalization of the data-driven method is the main limitation. In the convolutional neural network, we used a drop-out layer for inter-participant analysis. The drop-out layer helps the model to generalize better, particularly when the dataset is small and there is a high possibility of overfitting the training data. The convolutional layers of CNN have the advantage of automatic feature extraction and outperform neural networks. An important aspect of data-driven methods is how the dataset is divided into training and test data. For instance, if the data has been recorded for one person in a single session and then has been split to train and test, the reliability of the results will be doubtful. In this study, we have also reported the inter-participant results, which are a better representation of the robustness of the model. Nevertheless, the accuracy of the estimated values is lower for the inter-participant method compared to previous studies [27].

In this study, we used a single shoe-mounted accelerometer. Shoe-mounted accelerometers are common among runners and multiple commercialized foot-pods measure gait features. However, future work should optimize sensor placement for lower extremity tracking based on a single accelerometer. Although mounting sensors on the shank or thigh might be less practical compared to shoe-mounted sensors, the position and number of sensors on the shoe need to be optimized in future studies. It may be possible to obtain more information by mounting multiple tiny accelerometers on shoes. In this study, the position of the accelerometer on the shoe was selected empirically; however, mounting an accelerometer above the heel or placing it in the insole could provide better information. This study was a preliminary study that focused on training deep learning models for a single session. The repeatability of the method between days and between sessions should be investigated in future studies. The generalizability of machine learning models for in-field use is challenging and needs to be investigated in future work. The gait kinematics of runners during outdoor running differ from indoor running, which might create challenges when testing the machine learning model outdoors based on an indoor running test.

## Figures and Tables

**Table 1:** Layers of convolutional neural networks.

Index	Layer	Output Shape	Setting
0	Input	(60,4)	
1	Dropout		0.1
2	1D-Conv	(58,50)	ReLU
3	1D-Conv	(56,50)	ReLU
4	MaxPool	(28,50)	
5	1D-Conv	(26,100)	ReLU
6	1D-Conv	(24,100)	ReLU
7	Flatten	2400	ReLU
8	Dense	100	ReLU
9	Dense	3	Linear

**Table 2:** Average error and accuracy of estimated angles (SD) in intra-participant models among 10 participants. RMSE, root mean squared error; NRMSE, normalized root mean squared error.

	Hip	Knee	Ankle
R^2^	0.97 (0.00)	0.98 (1.30)	0.97 (1.59)
RMSE (deg)	2.3 (0.5)	3.4 (1.2)	1.8 (0.4)
NRMSE (%)	4.6 (0.1)	3.5 (0.1)	4.3 (0.1)

**Table 3:** Estimated and reference joint angles (SD) at discrete gait events in the intra-participant model. MAE, mean absolute error; DF, dorsiflexion; PF, plantarflexion.

	Hip	Hip	Knee	Ankle	Ankle	Ankle
	Peak Flexion	Peak Extension	Peak Flexion during Stance	Peak DF	Peak PF	PF/DF at Initial Contact
**Reference**	27.6 (7.3)	15.3 (3.9)	30.1 (6.4)	12.8 (3.6)	21.4 (5.0)	2.2 (3.1)
**Estimated**	28.3 (8.0)	15.5 (4.8)	30.2 (6.4)	13.1 (3.9)	20.9 (5.5)	2.2 (3.2)
**MAE**	2.4 (2.5)	1.5 (0.3)	1.2 (0.3)	1.0 (0.6)	1.0 (0.3)	1.0 (0.3)

**Table 4:** Average error and accuracy of estimated angles (SD) in the inter-participant models among 10 participants. RMSE, root mean squared error; NRMSE, normalized root mean squared error.

	Hip	Knee	Ankle
R^2^	0.84 (0.10)	0.93 (0.04)	0.78 (0.10)
RMSE (deg)	5.6 (2.2)	6.5 (2.1)	4.7 (1.6)
NRMSE (%)	9.9 (2.2)	6.5 (1.8)	11.1 (3.1)

**Table 5:** Estimated and reference joint angles at discrete gait events in the inter-participant model. MAE, mean absolute error; DF, dorsiflexion; PF, plantarflexion.

	Hip	Hip	Knee	Ankle	Ankle	Ankle
	Peak Flexion	Peak Extension	Peak Flexion during Stance	Peak DF	Peak PF	PF/DF at Initial Contact
Reference	27.6 (5.6)	15.6 (3.9)	30.0 (5.7)	13.1 (3.9)	21.0 (5.5)	2.4 (3.1)
Estimated	25.9 (3.0)	13.9 (1.4)	26.4 (2.4)	10.8 (2.0)	19.8 (4.1)	0.9 (1.5)
MAE	5.9 (3.3)	4.0 (1.5)	6.4 (4.6)	5.0 (2.3)	4.0 (2.5)	2.8 (1.3)

**Table 6:** Inertial-based methods for running kinematic estimation.

Ref.	Number of Sensors	Inter/Intra -Model	Method	Joint	RMSE
[23]	7 (Gyro + Acc)	Yes/No	Musculoskeletal modeling	Hip	8.7
				Knee	5.3
				Ankle	4.6
[27]	3 (Gyro + Acc + Mag)	Yes/Yes	Data-Driven	Knee	~4/<13
[20]	2 (Gyro + Acc)	Yes/No	Model-based	Knee	3.4
Ours	1 (Acc)	Yes/Yes	Data-Driven	Hip	2.3/5.6
				Knee	3.4/6.5
				Ankle	1.8/4.7
